# Chronic CRYPTOCHROME deficiency enhances cell-intrinsic antiviral defences

**DOI:** 10.1098/rstb.2023.0344

**Published:** 2025-01-23

**Authors:** Christine T. Major-Styles, Jack Munns, Aiwei Zeng, Michael Vanden Oever, John S. O'Neill, Rachel S. Edgar

**Affiliations:** ^1^ Department of Infectious Disease, Imperial College London, London SW7 2AZ, UK; ^2^ Francis Crick Institute, London NW1 1AT, UK; ^3^ MRC Laboratory of Molecular Biology, Francis Crick Avenue, Cambridge CB2 0QH, UK

**Keywords:** CRYPTOCHROME, circadian rhythms, virus, interferon, protein homeostasis, integrated stress response

## Abstract

The within-host environment changes over circadian time and influences the replication and severity of viruses. Genetic knockout of the circadian transcription factors CRYPTOCHROME 1 and CRYPTOCHROME 2 (*CRY1*^−/−^/*CRY2*^−/−^; CKO) leads to altered protein homeostasis and chronic activation of the integrated stress response (ISR). The adaptive ISR signalling pathways help restore cellular homeostasis by downregulating protein synthesis in response to endoplasmic reticulum overloading or viral infections. By quantitative mass spectrometry analysis, we reveal that many viral recognition proteins and type I interferon (IFN) effectors are significantly upregulated in lung fibroblast cells from CKO mice compared with wild-type (WT) mice. This basal ‘antiviral state’ restricts the growth of influenza A virus and is governed by the interaction between proteotoxic stress response pathways and constitutive type I IFN signalling. CKO proteome composition and type I IFN signature were partially phenocopied upon sustained depletion of CRYPTOCHROME (CRY) proteins using a small-molecule CRY degrader, with modest differential gene expression consistent with differences seen between CKO and WT cells. Our results highlight the crosstalk between circadian rhythms, cell-intrinsic antiviral defences and protein homeostasis, providing a tractable molecular model to investigate the interface of these key contributors to human health and disease.

This article is part of the Theo Murphy meeting issue ‘Circadian rhythms in infection and immunity’.

## 1. Introduction

For all infectious agents, the host environment changes substantially over time depending on prior exposure, physiological stress and endogenous circadian clocks. Most facets of the immune system exhibit circadian rhythmicity, including inflammation, antigen presentation, immune cell trafficking and function [1–5]. Experimental disruption of circadian clock components leads to inflammation and autoimmunity [1,6,7], whereas daily oscillations in systemic immune responses can account for time-of-day variation in infectious disease severity driven by immunopathology [8,9], but what about the establishment of infection? Initial time-of-day differences in production of new virus particles at the primary infection site can amplify as infection progresses and disseminates to other peripheral tissues [10,11]. Mucosal surfaces are continually bombarded with infectious threats and their basal state can determine the success or failure of incoming virus. Viruses are obligate intracellular pathogens so must also contend with host cell clocks in the epithelial and stromal cells they infect, which could regulate the innate defences generated locally in tissue that shape downstream systemic responses [12,13].

**Figure 1.**
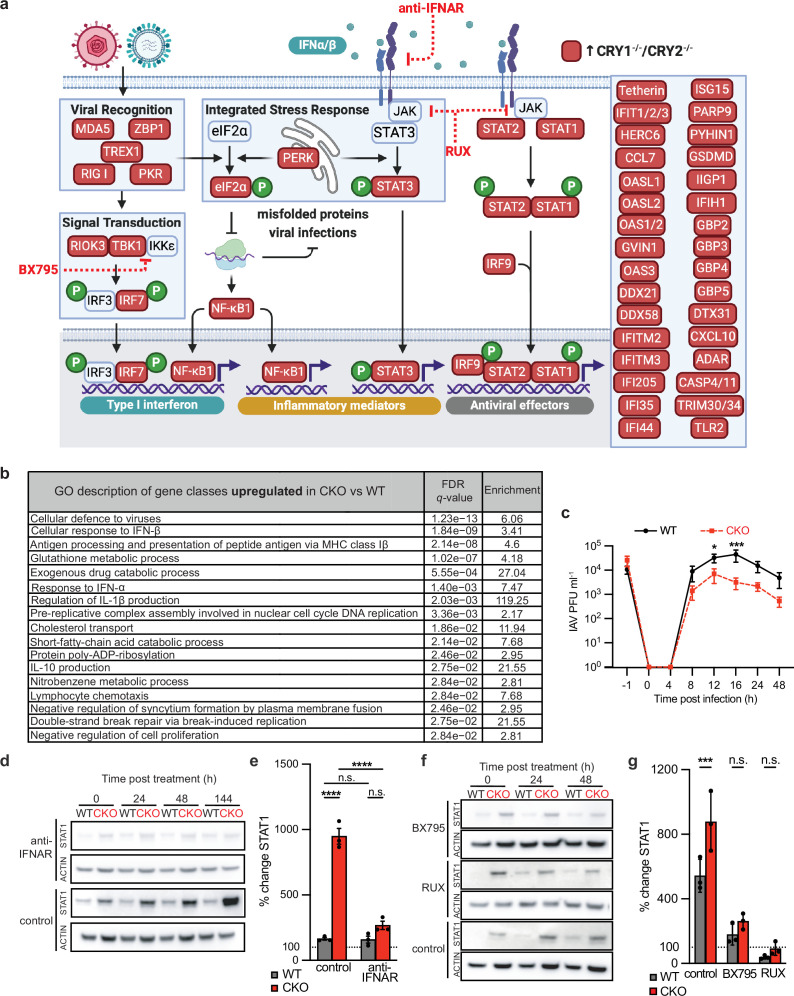
Aberrant constitutive type I IFN signalling in CRYPTOCHROME (CRY) knock-out (*CRY1*^*−/−*^*/CRY2*^*−/−*^; CKO) cells restricts virus growth. (*a*) Schematic representation of antiviral defence proteins significantly upregulated in CKO mouse lung fibroblasts compared with wild-type (WT) controls (created using BioRender). (*b*) Ranked gene ontology (GO) enrichment analysis was carried out using GOrilla on protein lists ordered by absolute fold change in abundance in CKO primary lung fibroblasts compared with WT controls (*n* = 4 biological replicates). For further data analysis please refer to Munns *et al*. [19]. FDR, false discovery rate. (*c*) Single-cycle influenza A virus (IAV) growth curves were performed on WT and CKO mouse lung fibroblasts (*n* = 3 biological replicates; mean ± s.e.m.). IAV-infected cells and supernatant were harvested at the stated times and infectious IAV particle levels were determined by plaque assay. PFU, plaque forming units. Two-way ANOVA (time × genotype): time *p < 0.05; genotype *p < 0.05; interaction *p < 0.05. Multiple comparison significance: *p < 0.05, ***p < 0.001. (*d,e*) Representative western blots and quantitative analysis of STAT1 abundance in WT and CKO mouse lung fibroblasts treated with anti-IFNAR1 monoclonal antibodies (anti-IFNAR) or isotype control antibody for 144 h. Summary graph shows percentage change in expression at 144 h relative to WT isotype control antibody at time = 0 h (*n* = 3 biological replicates; mean ± s.e.m.). Two-way ANOVA (time × treatment): time ****p < 0.0001; treatment ****p < 0.0001; interaction ****p < 0.0001. (*f,g*) Representative blots and quantitative analysis of STAT1 abundance in WT and CKO mouse lung fibroblasts treated with BX795, ruxolitinib (RUX) or dimethyl sulfoxide (DMSO) vehicle control for 48 h. Summary graph shows percentage change in expression at 48 h relative to WT DMSO vehicle control at time = 0 h (*n* = 3 biological replicates; mean ± s.e.m.). Two-way ANOVA (time × treatment): time **p < 0.01; treatment ****p < 0.0001; interaction *p < 0.05; n.s., not significant.

Circadian rhythms permeate cell biology and regulate most fundamental processes co-opted during viral replication, including transcription, translation and metabolism [10,14]. In addition, molecular clocks could influence cell-intrinsic antiviral responses that form the first line of defence against infection. These are predominantly driven by induction of type I or type III interferons (IFN) upon virus detection and the proteins encoded by >300 interferon-stimulated genes (ISGs), which inhibit virus replication in a myriad of different ways and shape subsequent inflammatory and adaptive immunity [12,15–18]. Many IFN signalling components are themselves IFN-inducible; cells constitutively secrete IFN-β to ensure homeostatic expression of ISGs that mediate viral recognition and signal transduction through the type I IFNα/β receptor IFNAR, for example, retinoic acid-inducible gene I (RIG-I) and signal transducer and activator of transcription (STAT) (figure 1*a*). This basal level of type I IFN is critical: too low and it compromises virus detection and innate responses, too high and it risks autoimmunity as type I IFN regulates inflammation [20].

The integrated stress response (ISR) is another key pathway that limits viral protein synthesis [21]. The ISR is activated by extrinsic and intrinsic factors such as virus infection and unfolded proteins in the endoplasmic reticulum (ER), respectively, that trigger phosphorylation and inactivation of eukaryotic translation-initiation factor 2 alpha (eIF2α) which shuts down translation and helps restore protein homeostasis (proteostasis). We recently found that genetic ablation of the transcription factors CRYPTOCHROME 1 and CRYPTOCHROME 2 (*C*RY*1*^−/−^/*C*RY*2*^−/−^; CKO) leads to chronic ISR activation [22]. CRY proteins are best known as transcriptional repressors integral to the canonical model of circadian timekeeping, whereby activators such as BMAL1 drive expression of clock-controlled genes (CCG), including *CRY1/2*, which then inhibit CCG transcription. The 24 h oscillations in mRNA levels resulting from this transcriptional–translational feedback loop (TTFL) were thought to generate accompanying changes in protein abundance and underpin rhythmic cellular physiology [23,24]. However, this linear causality has been challenged by multi-omic analyses that show poor correlation between the identities of rhythmic mRNAs and proteins [25–28]. CKO cells lack rhythmic gene expression yet at least as many proteins show circadian rhythms in their abundance and phosphorylation as in wild-type (WT) controls, but with extensive time-independent differences in (phospho)proteome composition between the two genotypes [22].

The activity of most proteins is governed post-translationally and there is growing recognition that TTFL components such as BMAL1, PERIOD and CRY have roles beyond transcriptional regulation. For example, BMAL1 is known to act as a translation factor while CRY also functions as an E3 ligase adaptor for the ubiquitin–proteasome system [29–31]. Mounting evidence suggests that circadian timekeeping might primarily function to enable temporal consolidation of proteome renewal and homeostasis, rather than by driving functional changes in protein abundance [22,23,32–34]. Loss of proteostasis and chronic stress that accompanies, and has long been thought to result from, circadian clock disruption could therefore impact upon cellular antiviral defences. We investigated this hypothesis using *ex vivo* lung fibroblast cells derived from WT and CKO mice.

## 2. Results

### (a) Aberrant constitutive type I interferon signalling in *CRY1*^*−/−*^*/CRY2*^*−/−*^ cells restricts virus growth

Several recent reports suggest a link between innate antiviral responses and the circadian clock but have only examined mRNA levels. For example, diurnal differences were observed in mouse skin ISG expression upon toll-like receptor 7 (TLR7) stimulation, with enhanced induction in *BMAL1*^−/−^ mice [35]. ISG transcripts were upregulated in a human lung cell line where BMAL1 was pharmacologically inhibited or silenced [36]. Whether this equates to increased levels of antiviral proteins and a restrictive environment remains to be seen. ISG-encoded proteins were not upregulated in primary lung fibroblasts from *BMAL1*^−/−^ mice and proteome analysis showed no enrichment for antiviral response pathways [11]. Constitutive IFN signalling and basal levels of ISG-encoded antiviral proteins often determine the response of individual cells to incoming virus particles for several reasons. First, replication of many viruses proceeds too quickly for induced ISGs to take effect. Second, most viruses restrict the translation of cellular proteins and this ‘host cell shut-off’ renders the magnitude of the ISG mRNA pool irrelevant [37,38]. Third, many viruses express proteins that antagonize IFN responses and blunt them as infection progresses [16]. We therefore directly compared WT and CKO fibroblast cell protein abundance and phosphorylation using quantitative mass spectrometry (figure 1*a*,*b*).

Genetic ablation of CRY proteins leads to significant upregulation of viral recognition and type I IFN induction pathway components, alongside downstream IFN signal transduction through JAK/STAT and a broad array of antiviral effector proteins in primary lung fibroblast cells (figure 1*a*). Consistent with our previous findings, we observed upregulation of key markers of ISR activation such as eIF2α abundance and Ser51 phosphorylation, along with the regulatory kinases PKR and PERK that inhibit eIF2α upon virus infection or ER overloading, respectively [22]. In line with previous reports, we also found significant upregulation of proinflammatory mediators such as Tyr705 phosphorylation of STAT3 and NF-κB signalling [6,22,39,40].

We next performed a ranked gene ontology (GO) analysis based on absolute fold change in protein abundance using GOrilla [41,42] (figure 1*b*). Examining proteins upregulated in CKO compared with WT cells, we found significant enrichment for GO terms associated with innate immune processes such as antiviral defence, type I IFN responses, antigen presentation and production of pro-inflammatory cytokines such as IL-1β. Previous proteomic analysis of WT and CKO cells sampled every 3 h over 72 h showed that these immune response proteins do not exhibit cell-autonomous circadian rhythms in their abundance for either genotype [22]. Performing ranked GO analysis on protein abundances averaged across all time points for these datasets revealed similar antiviral defence term enrichment for proteins upregulated at baseline in CKO cells compared with WT (electronic supplementary material, figure S1).

To determine whether the basal antiviral state we observe upon CRYPTOCHROME deletion can functionally restrict virus replication we assessed single-cycle influenza A virus (IAV) growth in WT and CKO primary lung fibroblast cells using the mouse-adapted H1N1 strain A/PR8/8/34 (figure 1*c*). In this infection assay, cells are simultaneously infected to examine influenza replication dynamics and average levels of virus production per cell. IAV replication is significantly slower in CKO cells, which consistently produce >10-fold lower amounts of new infectious particles compared with WT cells. CKO cells maintained a virus growth deficit upon exposure to a 10-fold higher level of infectious particles (electronic supplementary material, figure S2).

We then asked whether constitutive signalling through the type I IFN receptor IFNAR was required to maintain the higher levels of antiviral defence proteins observed with chronic CRY deficiency. We noticed that basal ISG levels for both WT and CKO cell monolayers start to increase after >24 h in culture without a medium change, which we speculate is due to cross-talk between IFN and ISR pathways as this is much more pronounced in CKO cells that have heightened sensitivity to proteotoxic stress [22] (figure 1*d*). For example, CKO cells showed >10-fold increase in STAT1 abundance following 144 h in culture compared with approximately 2-fold increase for WT cells (figure 1*e*). When cells were treated with an anti-IFNAR monoclonal antibody that blocks its activity, we observed no significant difference in STAT1 abundance between WT and CKO cells (figure 1*e*). From this, we conclude that reducing constitutive type I IFN signalling in CKO cells is sufficient to reverse their basal antiviral state.

What upstream and downstream pathways alter type I IFN activity in CKO cells? To address this question, we used specific inhibitors of TANK-binding kinase 1 (TBK1), which transduces virus recognition signals to induce type I IFN synthesis, and the STAT-interacting partner Janus kinase (JAK), which relays signals from IFNAR (figure 1*a*). As observed previously, STAT1 abundance increased in cells after 48 h in culture, approximately 2-fold for WT and >10-fold for CKO. Specific inhibition of either TBK1 by BX795 [43] or JAK by ruxolitinib [44] limited this increase and reversed the significant difference in STAT1 protein levels between WT and CKO cells (figure 1*f*,*g*). Collectively, these results indicate that the homeostatic ‘set point’ for constitutive type I IFN activity is higher in CKO cells, where an aberrant type I IFN feedback loop is driven by canonical TBK1 and JAK/STAT signalling pathways.

### (b) Loss of CRY proteins does not directly induce the altered type I interferon signature of CKO cells

The basal antiviral state of CKO cells can restrict virus growth, so we wanted to know the mechanism by which CRY protein deficiency leads to an activated type I IFN signature. One possibility is that TTFL transcriptional activators such as BMAL1 drive type I IFN and ISG expression, so the release of CRY-mediated transcriptional repression causes their upregulation in CKO cells. If this were the case, reciprocal downregulation of these antiviral proteins might be expected in BMAL1-deficient cells, which was not observed in prior proteomic analyses [11]. Genetic ablation of CRYs leads to an imbalanced proteome, with significantly higher cellular protein concentration in CKO compared to WT cells, and increased sensitivity to proteotoxic stress [22]. The loss of proteostasis that results from chronic CRY deficiency could indirectly affect constitutive type I IFN activity and generate the antiviral phenotype we observed in CKO cells.

To distinguish between these possibilities, we utilized a tool to transiently degrade CRY1 and CRY2 proteins; the putative CRY degrader C8. This small-molecule approach uses a CRY-binding small molecule KS15 conjugated to an E3 ligase and has been extensively validated, with comprehensive functional characterization of C8 performed in mouse fibroblasts [19]. Incubating cells with compound C8 efficiently depletes CRY protein to the limits of detection by western blot [19]. We compared the proteome composition of cells treated with C8 CRY degrader and dimethyl sulfoxide (DMSO) vehicle control for 24 h. Transient decrease in CRY protein abundance did not lead to ISG upregulation or significant enrichment for antiviral GO terms. Instead, we observed significant enrichment for DNA-binding pathways and CRY-interacting transcription factors, consistent with loss of CRY transcriptional repression.

**Figure 2.**
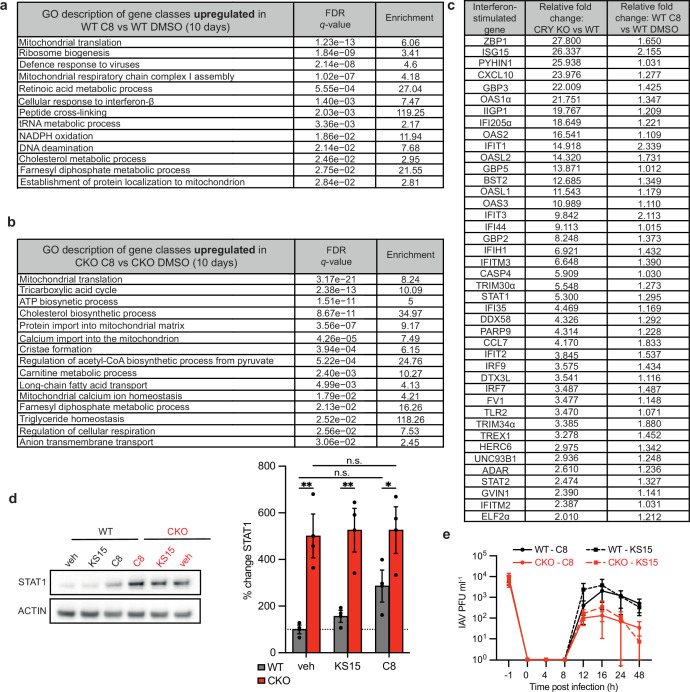
Loss of CRYPTOCHROME (CRY) proteins does not directly induce the altered type I interferon signature of CRY knock-out (*CRY1*^*−/−*^*/CRY2*^*−/−*^; CKO) cells. Ranked GO enrichment analysis was carried out using GOrilla on protein lists ordered by absolute fold change in abundance. Terms with a false discovery rate *q*-value < 0.05 were considered significant. Wild-type (WT) (*a*) and CKO (*b*) mouse fibroblasts were analysed after treatment with C8 or dimethyl sulfoxide (DMSO) vehicle control for 10 days (*n* = 2 biological replicates; *n* = 4 technical replicates). For further data analysis please refer to Munns *et al*. [19]. (*c*) Relative induction of interferon-stimulated genes (ISGs) in CKO cells compared with WT controls versus C8-treated WT cells compared with DMSO controls. KO, knockout. (*d*) Representative western blots and quantitative analysis of STAT1 abundance in WT and CKO cells treated with C8, KS15 control or DMSO vehicle control (veh) for 10 days. Summary graph shows percentage change in expression relative to WT DMSO vehicle control (*n* = 3 biological replicates; mean ± s.e.m.). One-way ANOVA multiple comparison significance: *p < 0.05; **p < 0.01. n.s., not significant. (*e*) Single-cycle influenza A virus (IAV) growth curves in WT and CKO mouse lung fibroblasts following 10 days of treatment with C8 CRY-degrader or KS15 CRY-binder control (*n* = 3 biological replicates; mean ± s.e.m.). IAV-infected cells and supernatant were harvested at stated times and infectious IAV particle levels were determined by plaque assay. PFU, plaque forming unit. Two-way ANOVA (time × treatment): time ****p < 0.0001; genotype *p* = 0.6554; interaction *p* = 0.9983.

We then assessed 16-plex tandem mass tag- (TMT-) quantitative mass spectrometry performed on primary lung fibroblast cells incubated with CRY-degrader C8 or DMSO control for 10 days, and analysed the results using a ranked GO analysis, as described in figure 1*b*. In WT cells, C8-mediated CRY depletion over this longer time frame resulted in upregulation of ISG abundance and significant enrichment for antiviral GO terms (e.g. ‘defence response to virus’) along with those involved in protein homeostasis (e.g. ‘ribosome biogenesis’), mitochondrial function (e.g. ‘mitochondrial translation’) and metabolism (e.g. ‘cholesterol metabolic process’) (figure 2*a*). The latter two are also enriched in CKO cells treated with C8 (figure 2*b*) and reflect non-specific ‘off-target’ effects of this compound [19].

Although the identities of ISGs significantly upregulated in CKO and CRY-depleted cells are the same, when we examined their absolute fold change in abundance it was far lower for the C8-treated cells (figure 2*c*). For example, STAT1 showed 5.3-fold increase in CKO compared with WT cells, whereas C8-treated WT cells showed a smaller 1.3-fold increase in STAT1 compared with control WT cells, which was harder to detect by western blot (figure 2*d*). These more modest changes in type I IFN signature observed after 10 days of CRY depletion were not sufficient to restrict virus growth: single-cycle IAV replication was not significantly different in WT cells treated with C8 CRY-degrader compared with KS15 control-treated cells (figure 2*e*).

### (c) Altered proteostasis and stress responses underpin differences in wild-type and CKO antiviral state

Comparison of CKO and C8-treated cells indicates that proteome imbalance and ISG upregulation develop gradually upon CRY protein depletion, independently from any acute loss of direct transcriptional repression. Several lines of evidence suggest that proteostasis can influence cell-intrinsic antiviral defences [45]. For example, pharmacological proteasomal inhibition can upregulate type I IFN, but the molecular links between ISR and IFN signalling pathways remain ill-defined. Similarly, rare human ‘type I interferonopathies’ are often caused by proteasomal mutations, but how these alter basal levels of IFN and generate autoimmunity is unclear [46].

**Figure 3.**
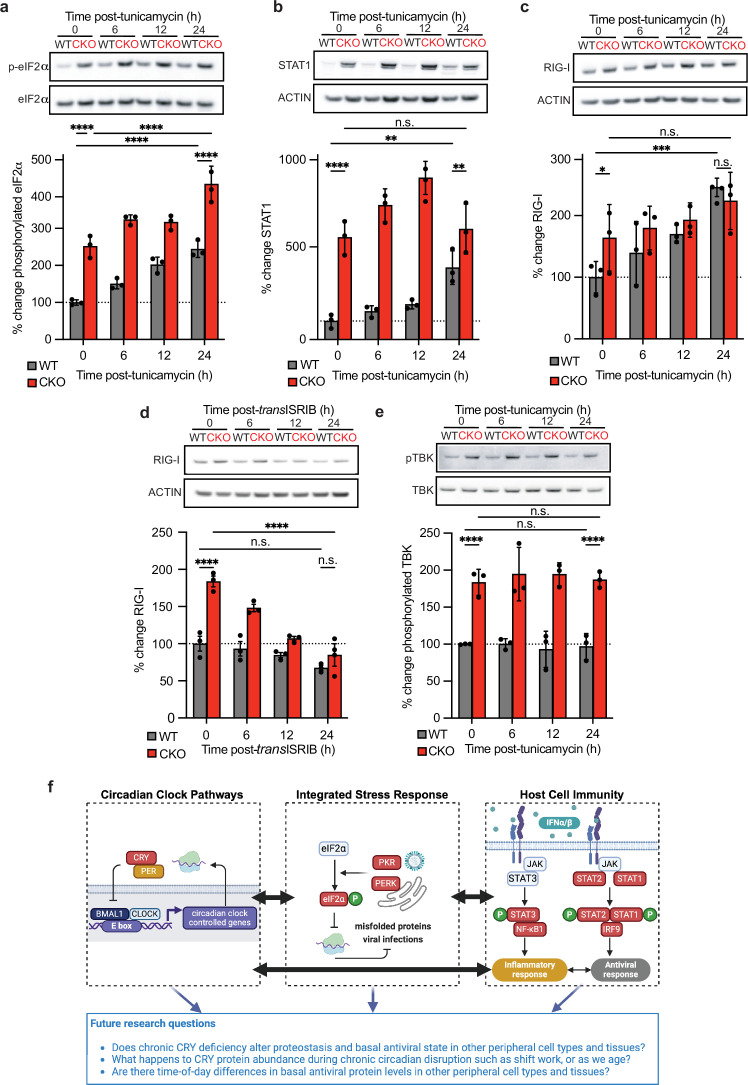
Altered proteostasis and stress responses underpin differences in wild-type (WT) and CRYPTOCHROME knock-out (*CRY1*^*−/−*^*/CRY2*^*−/−*^; CKO) antiviral state. Representative western blots and quantitative analysis of protein samples harvested from WT and CKO mouse lung fibroblasts treated with an integrated stress response (ISR) activator or inhibitor over 24 h (*n* = 3 biological replicates; mean ± s.e.m). (*a*) Phosphorylated eIF2α during tunicamycin treatment, (*b*) STAT1 during tunicamycin treatment, (*c*) RIG-I during tunicamycin treatment, (*d*) RIG-I during *trans*ISRIB treatment, and (*e*) phosphorylated TBK1 during tunicamycin treatment. Two-way ANOVA (time × treatment) multiple comparison significance: *p < 0.05; **p < 0.01; ***p < 0.001; ****p < 0.0001. (*f*) Summary illustration of the interplay between cell-intrinsic innate defence signalling and circadian clock pathways, and future research questions (created using BioRender).

We postulated that our antiviral CKO phenotype is caused by chronic proteotoxic stress: the proportion of phosphorylated eIF2α is significantly elevated in CKO cells compared with WT controls (figure 3*a*). We predicted that ISG abundance in WT cells would phenocopy that in CKO cells upon activation of the ISR by tunicamycin. This antibiotic blocks protein glycosylation, causing unfolded proteins to accumulate within the ER [47]. eIF2α phosphorylation increased upon tunicamycin treatment (figure 3*a*), confirming activation of the ISR. Tunicamycin increased the abundance of both STAT1 and RIG-I proteins as predicted, with WT levels of these ISGs mirroring CKO levels within 24 h post-treatment (figure 3*b*,*c*). Conversely, RIG-I abundance decreased when the ISR was inhibited by *trans*ISRIB treatment (figure 3*d*). *trans*ISRIB destabilizes the interaction between phosphorylated eIF2α and eIF2B that shuts off translation, and so blocks the ISR more effectively when phosphorylated eIF2α levels are high [48]. Consistent with this mode of action, *trans*ISRIB treatment decreased relative RIG-I abundance to a greater extent in CKO cells than WT cells.

Canonical TBK signalling did not drive the observed increase in ISG abundance upon activation of the ISR, as the proportion of phosphorylated TBK1 remained unchanged during tunicamycin treatment (figure 3*e*), so further research is required to identify the upstream signalling intermediates involved.

## 3. Discussion

CRY proteins are the central repressors of BMAL1 activity within the circadian TTFL circuitry so pathophysiological consequences of CRY deficiency are often ascribed to impairment of daily rhythms in gene expression [23,24,49]. By comparing the changes in proteome composition that arise from acute and chronic loss of CRY proteins, our research sounds a note of caution in attributing the diverse phenotypes of ‘clock gene’ mutants solely to TTFL disruption. Our findings support a model whereby CRY deletion results in proteotoxic stress and ISR activation which alters the basal antiviral state, as we can modulate WT and CKO ISG signatures with drugs that enhance or inhibit phosphorylated eIF2α activity (figure 3*f*). Why proteome imbalance develops upon loss of CRY proteins is an open question. CRY proteins could directly contribute to protein homeostasis through their reported role in proteasomal degradation: diverse CKO phenotypes mirror that of other E3 ligase adaptor knockout mice [22,23].

Crosstalk between type I IFN and the ISR remains enigmatic [45]. Both synergistic and antagonistic relationships have been described between different facets of cellular inflammatory, antiviral and stress responses, so the outcome of disrupting different clock components will depend upon how this alters the balance between these systems. CKO cells provide a tractable *in vitro* model to delineate how these major antiviral defences communicate and the consequences of perturbing their homeostatic equilibrium, and shed light on the mechanistic basis of type I interferonopathies [46].

Circadian rhythms are much less robust in CKO cells and mice [22,50]. Our research suggests that dysregulated circadian rhythms can impact infection susceptibility and cell-intrinsic immunity by compromising protein homeostasis. Examining how these pathways interact in and between other peripheral cell types targeted by viruses, such as epithelial cells, immune cells and hepatocytes, is now paramount (figure 3*f*) [10]. We previously found no evidence of cell-autonomous circadian rhythms in antiviral protein levels within primary mouse lung fibroblasts and must now determine if there are functional diurnal rhythms in constitutive type I IFN within peripheral tissues. Our findings indicate that only high-amplitude oscillations in basal ISG-encoded protein abundance will influence infection at mucosal surfaces.

Another outstanding question is what happens to homeostatic proteome renewal in models of chronic circadian dysregulation, such as shift work and as we age, in particular basal expression levels of clock components like BMAL1 and CRYPTOCHROME. Ageing is associated with inflammatory immunopathology, impaired circadian timekeeping and loss of proteostasis [51–54]. Further investigation into CRY protein abundance, ISR and antiviral state in experimental models of ageing and shift work is required to disentangle the causality of these interactions.

More broadly, we need to establish whether host biological time and ‘circadian hygiene’ can affect virus transmission. There are few animal models of transmission for clinically important respiratory infections such as influenza and SARS-CoV-2, each with substantial limitations [55]. Combining *in vitro* systems that model airborne pathogen transmission with human airway epithelial cultures or organoids may provide a viable alternative for future study [56–58]. Ultimately, incorporating chronotype and time-of-day considerations into human challenge trials is paramount if we are to understand the complex interaction between host circadian rhythms and viral infections.

## 4. Methods

### (a) Cell culture

Madin-Darby canine kidney (MDCK) cells were routinely cultured in 5% CO_2_ at 37°C in a humidified incubator. MDCK cells were cultured in Dulbecco’s modified Eagle medium (DMEM) supplemented with 100 U penicillin and streptomycin (pen/strep), 1× GlutaMAX (Gibco-Life Technologies) and 10% fetal calf serum (FCS; Gibco-Life Technologies). WT and CRYPTOCHROME knock-out (*CRY1^−/−^*, *CRY2^−/−^* and CKO) mouse lung fibroblasts (MFs) were cultured in DMEM supplemented with 100 U penicillin and streptomycin (pen/strep), 1× GlutaMAX (Gibco-Life Technologies) and 15% HyClone FetalClone III FCS. Adult MF cultures were established and maintained as per the protocol developed by Seluanov *et al.* [59]. Briefly, isolated MFs were initially grown at 3% O_2_ and 5% CO_2_ at 37°C, which enables indefinite culture of primary fibroblasts [60]. Prior to experimentation, cells were transferred to atmospheric oxygen for several passages (<10). MF cultures were used prior to senescence or crisis and retained characteristics of primary cells such as contact inhibition.

### (b) Quantitative mass spectrometry

#### (i) Experimental protocol

WT and CKO MFs (passages 16 and 14, respectively) [22] were grown to confluence in six-well plates (3.5 cm diameter) and maintained at 37°C throughout (except at treatment times) in standard medium containing 10% serum (FBS; 10270106, Gibco). Cells of each genotype were then treated with either 1 μM C8 or a vehicle treatment (DMSO to 0.01%) as follows: at the start of the experiment (day 1) the medium was aspirated, and cells were washed with 1 ml phosphate-buffered saline (PBS) and replaced with 1 ml medium as above supplemented with the compound or vehicle. Further identical medium changes were performed on three additional days (3, 6 and 9). All medium changes took place at the same time (within 1 h) on each respective treatment day. On day 10, cells were lysed and total protein was extracted. We generated four replicate samples for each condition (16 in total), with each replicate containing protein extracted from two independent plate wells.

#### (ii) Cell lysis and protein extraction

Cells were lysed at room temperature in 8 M urea buffer (8 M urea in 20 mM Tris, pH 8) containing 1 cOmplete™, EDTA-free protease inhibitor cocktail tablet per 10 ml buffer (Roche); the medium was aspirated, and cells were washed with 2 ml PBS and then incubated in 100 μl urea buffer for at least 15 min. Cells were then scraped, collected, snap-frozen in liquid nitrogen and stored at −80°C.

Lysates were later thawed on ice, given four rounds of 30 s sonication (Diagenode Bioruptor^®^ Plus), and centrifuged for 15 min at 4°C at 16 000*g*, and the supernatant was collected. Extracted protein was then quantified using a Pierce™ BCA Protein Assay Kit (ThermoFisher Scientific) following the manufacturer's protocol. Samples were stored at −80°C.

#### (iii) Sample digestion, TMT (tandem mass tag) labelling and fractionation

Protein samples (50 µg) were diluted with 4 M urea and reduced with 5 mM dithithreitol (DTT) at 56°C for 30 min, then alkylated with 10 mM iodoacetamide in the dark at room temperature for 30 min. Excess iodoacetamide was quenched by the addition of 5 mM DTT for 10 min. The samples were then diluted to 2 M urea and digested with Lys-C (Promega) at a ratio of 1 : 50 enzyme : protein for 4 h at 25°C. Next, the samples were further diluted to 1.5 M urea, and trypsin (Promega), at a ratio of 1 : 60 enzyme : protein, was added and incubated overnight at 30°C. Digestion was stopped by the addition of formic acid (FA) to a final concentration of 0.5%. Any particulate matter was removed by centrifugation at 16 000*g* for 5 min. Supernatants were desalted using home-made C18 stage tips (3 M Empore) filled with 1.2 mg of Oligo R3 resin (Thermo Scientific). Stage tips were equilibrated with 80% acetonitrile (MeCN)/0.5% FA followed by 0.5% FA. Bound peptides were eluted with 30–80% MeCN/0.5% FA and lyophilized.

Dried peptide mixtures from each condition were resuspended in 30 µl of 200 mM HEPES, pH 8.5. Isobaric labelling of the peptides was performed using TMTpro 18-plex reagents (Thermo Fisher Scientific); 15 µl reagent, reconstituted in anhydrous MeCN, was added and samples incubated at room temperature for 1 h. The labelling reaction was then quenched by incubation with 5% hydroxylamine for 30 min. TMT-labelled peptides were pooled into a single sample and desalted using stage tips as described above.

High-pH fractionation of labelled peptides was performed by offline high-pressure liquid chromatography using an XBridge BEH130 C18, 5 µm, 2.1 × 150 mm (Waters) column with XBridge BEH C18 5 µm Van Guard Cartridge, connected to an Ultimate 3000 Nano/Capillary LC System (Dionex). Peptides were separated with a gradient of 1–90% B (A: 5% MeCN/10 mM ammonium bicarbonate, pH 8; B: MeCN/10 mM ammonium bicarbonate, pH 8 (9 : 1)) in 1 h at a flow rate of 250 µl min^−1^. Eluted peptides were collected at 1 min per fraction; 54 fractions were collected and concatenated into 18 fractions and lyophilized. Dried peptides were resuspended in 1% MeCN/0.5% FA, desalted using C18 stage tips and partially dried down by vacuum centrifugation.

#### (iv) Mass spectrometry analysis

The fractionated peptides were analysed by liquid chromatography–mass spectometry/mass spectrometry (LC-MS/MS) using a fully automated Ultimate 3000 RSLC nano System (Thermo Fisher Scientific) fitted with a 100 μm × 2 cm PepMap100 C18 nano trap column and a 75 μm × 25 cm nanoEase M/Z HSS C18 T3 column (Waters). Peptides were separated using a binary gradient consisting of buffer A (2% MeCN, 0.1% FA) and buffer B (80% MeCN, 0.1% FA), at a flow rate of 300 nl min^−1^ . Eluted peptides were introduced directly through a nanoFlex ion source into an Orbitrap Eclipse mass spectrometer (Thermo Fisher Scientific). The mass spectrometer was operated in real-time database search (RTS) with synchronous-precursor selection (SPS)-MS3 analysis for reporter ion quantification.

MS1 spectra were acquired using the following settings: resolution = 120,000 ; mass range = 400–1400 *m*/*z*; AGC target = 4e5; MaxIT = 50 ms; dynamic exclusion = 60 s. MS2 analysis was carried out with HCD activation, ion trap detection, AGC = 1e4; MaxIT = 50 ms; NCE = 33% and isolation window = 0.7 *m*/*z*. RTS of MS2 spectrum was set up to search the UniProt *Mus musculus* proteome, with fixed modifications cysteine carbamidomethylation and TMTpro 16-plex at N-terminal and Lys residue. Met-oxidation was set as variable modification. Missed cleavage = 1 and maximum variable modifications = 2. In MS3 scans, the selected precursors were fragmented by HCD and analysed using the Orbitrap with settings as follows: isolation window = 1.3 *m*/*z*; NCE = 55; Orbitrap resolution = 50,000 ; scan range = 110–500 *m*/*z*; MaxIT = 200 ms; AGC = 1e5. The acquired raw files from LC-MS/MS were processed using MaxQuant [61] with the integrated Andromeda search engine (v. 1.6.17.0). MS/MS spectra were quantified with reporter ion MS3 from TMTpro 18-plex experiments and searched against the UniProt *Mus musculus* proteome FASTA database (downloaded on November 2020). Carbamidomethylation of cysteines was set as fixed modification, while methionine oxidation and protein N-terminal acetylation were set as dynamic modifications. Tryptic digestion up to two missed cleavages was allowed.

#### (v) Data analysis

The MaxQuant output file was first processed with Perseus software (v. 1.6.15.0); data were filtered to remove identifications from reverse database, identifications with modified peptide only, and common contaminants. Data were exported to Microsoft Excel, and proteins with zero values in all samples were removed. Protein abundance was then normalized to a scaling factor such that the sums of each TMT channel were equivalent (scaling to the sum of the lowest channel). Data were filtered to include only proteins with abundance >0 in at least 1 WT vehicle-treated sample.

Absolute fold change was calculated from these values, taking the average abundance for each protein in vehicle-treated CKO and WT vehicle-treated conditions and dividing the larger by the smaller. For abundance comparisons, data were transformed (log_2_(abundance value + 1)) and significant differences were calculated using *t*-tests (with Benjamini–Hochberg-corrected *q*-value <0.05 being taken as significantly different). For GO analysis, enriched terms were identified using GOrilla [41,42], comparing ranked lists of all 7559 detected proteins in order of absolute fold change in abundance compared with stated control condition. We used REVIGO to reduce redundant terms in figures 1 and 2 and electronic supplementary material, S1 [62], along with further manual curation.

### (c) Interferon pathway inhibitor treatment of mouse fibroblasts

WT and CKO lung MFs were seeded for confluence in six-well plates before being treated with IFN pathway inhibitors (table 1). Cells were treated in 1 ml full growth medium at the concentrations described in table 1 and protein samples were harvested between 0 and 144 h post-treatment, as described. Upon harvesting, medium was aspirated, and cells were washed with 2 ml cold 1× PBS before addition of 400 µl of RIPA lysis buffer (50 mM Tris pH 7.5, 0.1% sodium docdecyl sulfate (SDS), 5 mM EDTA, 150 mM NaCl, 1% X-100 Triton) supplemented with PhosSTOP (Merck Life Science) and cOmplete protease inhibitor cocktail (Merck Life Science) as per manufacturer’s instructions. After 30 min incubation on ice, cells were scraped and liquid removed to an Eppendorf tube before centrifugation for 10 min at 10 000 rpm at 4°C, then 300 µl of supernatant was removed to a fresh tube. Protein samples were reduced in 50 mM TCEP (tris(2-carboxyethyl)phosphine) in 4× Bolt LDS Sample Buffer (Life Technologies) and heated for 10 min at 70°C and returned to ice before western blotting analysis.

**Table 1.** Summary of drugs used to treat wild-type and CKO mouse lung fibroblasts.

drug treatment	concentration	vehicle/control	manufacturer
tunicamycin	500 nM	DMSO	Bio-Techne
*trans*ISRIB	100 nM	DMSO	Bio-Techne
IFNAR monoclonal antibody	1 µg ml^−1^	isotype control antibody	ThermoFisher Scientific
BX795	6 µM	DMSO	Stratech
ruxolitinib	1 µM	DMSO	VWR International

### (d) Western blotting

For western blot analysis proteins were separated on pre-cast NuPAGE 4–12% Bis-Tris 1 mm polyacrylamide gels at 150 V for 70 min in a NuPAGE XCell4 SureLock^TM^ electrophoresis system, before transfer to nitrocellulose membranes using iBlot^TM^ transfer stacks in the iBlot^TM^ 2 transfer system for 7 min at 20 V. All reagents were purchased from Life Technologies. Membranes were then blocked in 5% BSA : TBST (Tris-buffered saline supplemented with 0.1% Tween) for 1 h at RT. Membranes were then incubated with the respective antibodies (detailed in table 2) in 5% BSA: TBST overnight at 4°C. After incubation, membranes were washed four times for 15 min in TBST then incubated with secondary horseradish peroxidase (HRP)-conjugated antibodies for 1 h at room temperature before washing as before. Proteins were visualized on an Azure c600 system using Immobilon reagent (Millipore). Densitometry analysis was performed using ImageJ.

**Table 2.** Summary of antibodies used for protein analysis.

antibody	dilution	manufacturer
anti-mouse HRP	1 : 10 000	Cell Signaling
anti-rabbit HRP	1 : 10 000	Cell Signaling
beta-actin	1 : 1000	Santa Cruz Biotechnology
STAT1	1 : 1000	Cell Signaling
RIG-I	1 : 1000	Cell Signaling
TBK1	1 : 1000	Cell Signaling
pTBK1	1 : 1000	Cell Signalling

### (e) Virus production and titration

Influenza A/PR8/8/34 (H1N1) was propagated by infection of confluent MDCK cells in serum-free DMEM medium (SFM; DMEM supplemented with pen/strep and GlutaMAX) with 1 µg ml^−1^ *N*-tosyl-ʟ-phenylalanyl chloromethyl ketone (TPCK)-treated trypsin (Worthingtons Bioscience). For quantification of virus titre, IAV was serially diluted in SFM before being incubated with MDCK cells grown to confluence in six-well plates for 1 h at 37°C. Input virus was removed by aspiration and 3 ml of overlay was added (SFM containing 0.14% bovine serum albumin (BSA), 0.8% Avicel (FMC BioPolymer), 1 mg ml^−1^ TPCK-treated trypsin). Cells were incubated for 3 days before fixing in 8–10% formalin/PBS and staining with 0.1% toluidine blue (Sigma Aldrich) and viral plaque-forming units (PFU) were quantified.

### (f) Viral growth curves

Single-cycle 48 h IAV growth curves were run on WT and CKO lung fibroblasts. For growth curves, MFs were seeded for confluence in six-well plates and each condition was performed in triplicate. Medium was removed and lung MF cells were washed with 2 ml PBS before incubation with 1 ml SFM containing 0.05 MOI (multiplicity of infection) IAV with 1 mg ml^−1^ TPCK-treated trypsin for 1 h at 37°C. Virus input was harvested prior to incubation. Following incubation, input virus was aspirated and cells were briefly washed with 1 ml HCl acid wash (10 mM HCl, 150 mM NaCl pH 3) before 3× PBS washes and then addition of 1 ml SFM. Virus was harvested at described time points by scraping of the cells and harvesting of cells and supernatant. Virus sample titres were then quantified in parallel as described above. For CRY-degrader growth curve experiments, cells were treated with C8, KS15 or DMSO for 10 days prior to IAV infection.

### (g) Cell viability measurements

Viability of WT and CKO lung fibroblasts undergoing drug treatment was determined through alamarBlue live-cell staining (ThermoFisher) as per the manufacturer’s instructions. Briefly, cells were seeded in 96-well plates 24 h prior to drug treatment at concentrations noted in table 1 and treated for 48 h before the addition of alamarBlue. Resulting fluorescence from live viable cells was analysed through FLUOstar Omega plate reader (BMG Labtech).

### (h) Statistics

Data were analysed in GraphPad Prism 10.4.1 and summary data are presented as mean ± standard error of the mean (s.e.m.) alongside individual replicates. Biological and technical replicates and statistical analyses are reported in the figure legends. For each biological replicate, data were normalized by the sum of all data points within that replicate as outlined by Degasperi *et al.* [63]. Conventional parametric statistical tests were utilized because the types of measurements performed tend to approximate a Gaussian distribution after normalization.

## Data Availability

Proteomics data and influenza growth curve titres are provided as elctronic supplementary material, figure S1 [64]. Scripts and processed data are available on GitHub [65], and mass spectrometry data have been deposited to the ProteomeXchange Consortium [66] through the PRIDE [67] partner repository with the dataset identifier PXD019499 (http://www.ebi.ac.uk/pride/archive/projects/PXD019499). Western blots are available on request.
